# Obesity and the risk of late mortality after aortic valve replacement with small prosthesis

**DOI:** 10.1186/1749-8090-8-174

**Published:** 2013-07-15

**Authors:** Biao Wang, Hongyang Yang, Shuming Wu, Guangqing Cao, Hongling Yang

**Affiliations:** 1 Department of Cardiovascular Surgery Qilu Hospital, Shandong University, Jinan, China

**Keywords:** Aortic valve replacement, Small aortic root, Obesity, Body mass index

## Abstract

**Background:**

Whether obesity is related to late mortality with implantation of small aortic prosthesis remains to be clarified. This study was aimed to evaluate the effect of obesity on late survival of patients after aortic valve replacement (AVR) with implantation of small aortic prosthesis (size ≤ 21 mm).

**Methods:**

From January 1998 to December 2008, 307 patients in our institution who underwent primary AVR with smaller prostheses survived the 30 days after surgery. Patients were defined as normal if body mass index (BMI) was < 24 kg/m^2^, as overweight if BMI 24–27.9 kg/m^2^, and as obese if BMI ≥ 28 kg/m^2^. Data of New York Heart Association (NYHA) functional classification, left ventricular ejection fraction (LVEF), effective orifice area index (EOAI), and left ventricular mass index (LVMI) of the patients collected at the 3rd month (M), 6th M, 1st year (Y), 3rd Y, 5th Y, 8th Y after operation respectively.

**Results:**

By multivariable analysis, obesity was an independent factor of late mortality (hazard ratio [HR]: 1.62; P = 0.01). The obesity and overweight group had more poor survival (p < 0.001) and higher proportion of NYHA class III/IV (p < 0.01) compared with the normal group. Lower EOAI and higher LVMI were found in obesity and overweight group, but we saw no significant difference about LVEF among the three groups.

**Conclusions:**

Obesity was associated with increased late mortality of patients after AVR with implantation of small aortic prosthesis. Being obese or and overweight may also affect the NYHA classification, even in the longer term. EOAI should be improved where possible, as it may reduce late mortality and improve quality of life in obese or overweight patients.

## Background

The aortic annulus of Chinese individuals is typically smaller than that of people of European descent, and thus our hospital in china treats many patients who have a small aortic annulus in aortic valve replacement (AVR). Techniques developed to expand the annulus [1-3] were rarely applied in clinical circumstances because these techniques may increase the complexity and risk in an operation [4,5]; most surgeons prefer to use a small aortic prosthesis instead of expanding the annulus. Yet the use of a small aortic prosthesis may be associated with obstruction of left ventricular output, resulting in a higher transvalvular gradient and patient-prosthesis mismatch (PPM) [6]. Studies [7,8] have demonstrated that mortality was higher in patients receiving a small aortic prosthesis; Further complicating the issue, there was a rapid increase in the obese population in recent decades, and obesity has become an important factor that seriously affects the health of Chinese people [9]. However, it is unclear whether obesity is related to later mortality in AVR. Here we report 307 patients from a single center in China who underwent AVR with small prostheses (size ≤ 21 mm); our long-term follow-up study investigated if obesity was an independent predictor of long-term mortality for these patients after AVR.

## Methods

### Patients

From January 1998 through December 2008, a total of 328 patients underwent primary AVR (size ≤ 21 mm) at the Department of cardiovascular surgery of Qilu Hospital, Shandong University, Jinan, China. 21 (6.4%) of the 328 patients who died within 30 days following surgery were excluded, so the study population was composed of 307 patients (mean age 56 ± 12.6), 58.3% (n = 179) are females. Etiology was rheumatic disease in 44.3% (n = 136) patients, congenital bicuspid valve in 25.5% (n = 78) patients, degenerative calcific disease in 22.4% (n = 69) patients, and endocarditis in 7.8% (n = 24) patients. 40.7% (n = 125) received bioprosthesis. Preoperative coronary angiography was performed in all patients aged ≥ 50 years old and in all patients with angina or in whom coronary artery disease was suspected on a clinical basis. 17.5% (n = 54) underwent concomitant coronary artery bypass graft surgery (CABG). The study was approved by the ethics review committee for human studies at the School of Medicine, Shandong University.

### Data collection

Pre-operative and operative data were prospectively collected and validated (Table 1). Follow-up information was available in all 307 patients. Most (68.4%, n = 210) were followed up regularly at our institution; telephone, E-mail, post, or out-patient interviews (29%, n = 89) were conducted with patients or first-degree relatives, to confirm mortality and get related data including weight, height, symptom, capacity for action and results of Two-dimensional and Dopper echocardiography; mortalities among the remaining patients (1.8%, n = 8) were determined using national death and survival data registry.

**Table 1:** Baseline pre-operative and operative data of total study groups in patients who survive the first postoperative month after AVR

	**BMI < 24**	**24 ≤ BMI ≤ 27.9**	**BMI ≥ 28**
	**n = 185**	**n = 94**	**n = 28**
Age, y	56 ± 11.5	54 ± 14.7	57.8 ± 10.3
Sex (female),n (%)	116 (64.4)	44 (47.3)*	19 (67.8)†
Body surface area,m^2^	1.72 ± 0.32	1.78 ± 0.21*	1.86 ± 0.28*†
NYHA functional class, n (%)			
III	85 (45.9)	36 (38.3)	13 (46.4)
IV	66 (35.7)	28 (29.8)	8 (28.6)
LVEF, %	49.6 ± 9.2	52.7 ± 7.8	51.8 ± 8.1
Pathology, n (%)			
Stenosis	64 (34.6)	26 (27.6)	8 (28.6)
Insufficiency	20 (10.8)	12 (12.8)	6 (21.4)*†
Mixed	101 (54.6)	56 (59.6)	14 (50)
Diabetes mellitus, n (%)	26 (14.1)	20 (21.2)*	8 (28.6)*†
Systemic hypertension, n (%)	41 (22.1)	28 (29.8)*	13 (46.4)*†
Chronic lung disease, n (%)	21 (11.4)	15 (15.9)*	6 (21.4)*†
AF, n (%)	75 (40.5)	28 (29.8)*	9 (32.7)*
Operative data			
Concomitant CABG	30 (16.2)	18 (19.1)*	6 (21.4)*†
EOAI,cm^2^/m^2^	1.07 ± 0.12	0.96 ± 0.08*	0.81 ± 0.05*†
CPB time, min	100 ±31	104 ± 42	112 ± 28*†
Mechanical prosthesis, %	126 (68.5)	54 (57.4)	16 (57.1)

*significant difference from normal (BMI < 24) group; † significant difference from overweight (24 ≤ BMI ≤ 27.9) group.

Abbreviations: *AVR* Aortic valve replacement, *BMI* Basic mass index, *NS* Not significant, *NYHA* New York Heart Association, *LVEF* Left ventricular eject fraction, *AF* Atrial fibrillation, *CABG* Coronary artery bypass graft, *EOAI* Effective orifice area index, *CPB* Cardiopulmonary bypass.

### BMI definition

We adopted the most commonly used BMI classification system for adults in China [10]. Patients were classified as normal weight if BMI was < 24 kg/m^2^, as overweight if BMI 24–27.9 kg/m^2^, and as obese if BMI ≥ 28 kg/m^2^. The cohort was divided into 3 groups according to BMI: normal weight (n = 185, 60.2%), overweight (n = 94, 30.6%), and obese (n = 28, 9.2%).

### Statistical methods

 Pre-operative and operative data are expressed as mean ± SD or percentages. Repeated analysis of variance (ANOVA) measures were used to test the significance of changes in data in different groups of the study; the Sheffe post-hoc test was utilized for multiple comparisons. The chi-square analysis or Fisher exact test was used to compare categorical data. Cumulative probability of survival was analyzed by constructing Kaplan-Meier curves among patients in three groups, and inter-group comparisons were performed using the long-rank test. Cox proportional-hazards regression models were used to determine whether overweight and obesity were associated with survival after AVR. The related variables were entered into the Cox analyses of observed survival. Significant (P ≤ 0.1) variables on univariate analysis were reentered in a multivariable model examining predictors of survival. A propensity score was calculated using a logistic regression analysis that identified variables, that may be associated with BMI. Variables included in the logistic regression analysis were: sex, age, body surface area (BSA), diabetes mellitus, systemic hypertension, chronic lung disease, EOAI, and cardiac pulmonary bypass (CPB) time. The propensity score was then incorporated into subsequent proportional-hazards models. Results were expressed as the hazard ratio (HR) and 95% of confidence interval (CI). All statistical analyses were carried out using SPSS 17.0 (SPSS, Chicago, IL).

## Results

In this study, normal BMI (< 24 kg/m^2^) was present in 62.5% of the patients (n = 185), overweight BMI (24–27.9 kg/m^2^) presented in 30.6% (n = 94) and obesity BMI (≥ 28 kg/m^2^) in 9.2% (n = 28). Pre-operative and operative data are shown in Table 1. Compared with the normal group, those patients in overweight and obesity groups had a smaller EOAI, a larger BSA, lower prevalence of AF, and a higher prevalence of diabetes mellitus, systemic hypertension, chronic lung disease, as well as concomitant coronary artery bypass grafting (CABG). The obesity group displayed a higher proportion of female patients than the overweight group, and predominant insufficiency was more frequent and CBP time longer in obesity group than the other two groups. We found no significant difference in the variables of age, NHYA class and prosthesis type (biological or mechanical) among three groups.

### Impact of BMI on morality

**Figure 1:**
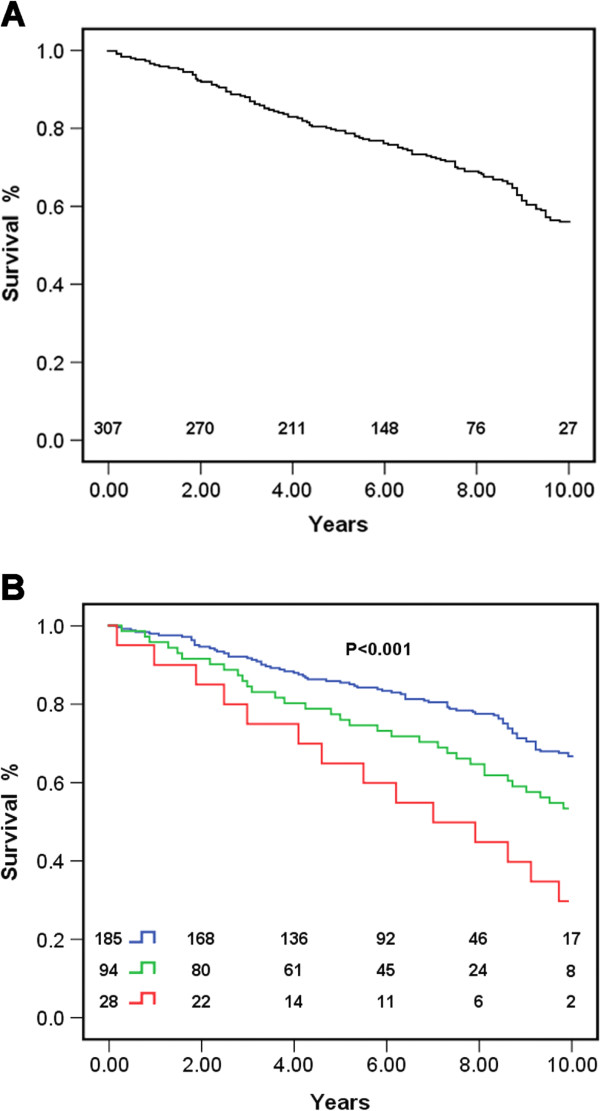
**Overall survival and separate survival of three groups divided by body mass group (BMI).** Blue indicates normal group (BMI < 24 kg/m^2^); green indicates overweight group (BMI 24–27.9 kg/m^2^); red line indicates obesity group (BMI ≥ 28 kg/m^2^). **A** Overall survival %. **B** Separate suvival %.

Mean overall follow-up duration was 4.7 ± 2.8 years (median, 4.1 years; range: 0.2-10 years). 68 deaths were observed during follow-up. The overall survival rate was 81.5% ± 2.6% at 5 years and 54.3% ± 1.4% at 10 years (Figure 1A). For obese patients, 5-year survival (65.1% ± 1.8%) and 10-year survival (31.6% ± 1.4%) were significantly lower than those of patients with normal BMI (5-year survival: 85.2% ± 3.6%; 10-year survival: 65% ± 2.2%; P = 0.007; Figure 1B). The obese group also had significantly lower survival than the overweight group (5-years survival: 74.8% ± 3.1%; 10-years survival: 52.2% ± 2.0%; P = 0.01). Difference in survival between the normal and overweight groups was also statistically significant (P = 0.03), with the overweight group having a lower survival rate.

### Predictors of mortality

According to univariate analysis (Table 2), the predictors of late post-operative mortality were old age, poor cardiac function, reduced LV ejection fraction (LVEF), suffering from diabetes, hypertension, chronic lung disease and atrial fibrillation (AF), concomitant CABG, use of a mechanical prosthesis, EOAI (≤0.85 cm^2^/m^2^), and obesity, with obesity presenting a higher level of statistical significance (HR: 1.84; 95% CI: 1.46 to 2.26; P < 0.001). Overweight also tended to be associated with higher mortality by univariate analysis (HR: 0.92; 95% CI: 0.71 to 1.15; P =0.01). For multivariate analysis (Table 2), after adjustment of the variables with a p ≤ 0.1 on univariate analysis, the following variables were rendered not independently associated with long-term survival: poor cardiac function, concomitant CABG, and use of a mechanical prosthesis; overweight also failed to be an independent predictor (HR: 0.64; 95% CI: 0.50 to 0.81; P =0.45), but obesity remained significantly associated with increased mortality (HR: 1.60; 95% CI: 0.32 to 1.91; P =0.008). After further adjustment for propensity score, the following variables were still independent predictors: old age (HR: 0.21; 95% CI: 1.04 to 1.10; P < 0.001), reduced LVEF (HR: 1.28; 95% CI: 1.09 to 1.51; P < 0.001), suffering from diabetes (HR: 1.02; 95% CI: 0.75 to 1.32; P =0.01, hypertension (HR: 1.01; 95% CI: 0.79 to 1.33; P < 0.001), chronic lung disease (HR: 1.30; 95% CI: 1.03 to 1.60; P = 0.003), EOAI (HR: 1.26; 95% CI: 1.09 to 1.44; P = 0.005) and obesity (HR: 1.62; 95% CI: 1.33 to 1.93; P = 0.01).

**Table 2:** Results of univariate and multivariate analyses based on observed late (deaths within 30 days of aortic valve replacement excluded) survival using the cox regression

	**Univariate analysis**		**Multivariate analysis 2**	
	**p Value**	**HR (95% CI)**	**p Value**	**HR (95% CI)**
Age	p < 0.001	1.34(1.12-1.58)	**p < 0.001**	**1.21(1.04-1.10)**
Sex	0.08	0.92(0.72-1.14)	0.21	0.89(0.84-0.95)
Body surface area	NS	1.34(1.14-1.53)	0.75	1.29(1.10-1.50)
NYHA functional calss III/IV	p < 0.001	1.25(1.04-1.47)	0.48	1.22(1.01-1.43)
LVEF	p < 0.001	1.58(1.12-2.06)	**p < 0.001**	**1.28(1.09-1.51)**
Predominant AS	0.05	1.11(0.84-1.40)	0.43	1.04(0.86-1.24)
Diabetes mellitus	p < 0.001	1.21(1.02-1.12)	**0.01**	**1.02(0.75-1.32)**
Systemic hypertension	p < 0.001	1.43(0.14-1.78)	**p < 0.001**	**1.20(0.87-1.53)**
Chronic lung disease	p < 0.001	1.39(1.20-1.63)	**0.003**	**1.30(1.03-1.60)**
AF	p < 0.001	1.27(1.04-1.56)	0.12	1.16(0.89-1.48)
Operative data				
Concomitant CABG	0.004	1.10(0.96-1.26)	0.11	1.02(0.90-1.15)
EOAI ≤ 0.85 cm^2^/m^2^	p < 0.001	1.42(1.12-1.73)	**0.005**	**1.26(1.09-1.44)**
Mechanical prosthesis	0.03	1.17(0.94-1.42)	0.08	0.88(0.65-1.10)
24 ≤ BMI ≤ 27.9	0.01	0.92(0.71-1.15)	0.34	0.68(0.50-0.92)
BMI ≥ 28	p < 0.001	1.84(1.46-2.26)	**0.01**	**1.62(1.33-1.93)**
Propensity score	0.75	1.51(1.18-1.94)	0.53	1.73(1.34-2.12)

**Bold** indicates statistical significance on multivariate analysis. Analysis 1: without adjustment for propensity score. Analysis 2: with adjustment for propensity score. *CI*, Confidence interval; *HR*, Hazard ratio; *AS*, Aortic stenosis; *BMI*, Body mass index; other abbreviations as in Table 1.

## Discussion

Here we reported the long-term follow-up with patients who underwent aortic prosthesis replacement with smaller prosthesis (size ≤ 21 mm) in china. A notable finding was produced that obesity (BMI ≥ 28 kg/m^2^) was an independent predictor of late mortality in patients undergoing AVR with small prosthesis. Obesity group and overweight group had poor survival and higher proportion of NYHA Function III/IV compared to normal group.

### Review of previous studies

There have been some different opinions in the published reports about the impact of BMI on outcomes after AVR, but specialized research on AVR with small prosthesis was scarce. Several studies demonstrated that BMI is an independent predictor of mortality and clinic events after AVR. Parwis [11] revealed obesity as an independent predictor of hospital and longer mortality in patients who underwent valve surgery. William [12] studied patients having AVR for AS with or without concomitant coronary artery bypass grafting and revealed a better survival in patients with low BMI compared to patients with higher BMI. On the other hand, others failed to found a significant effect of BMI on post-operative outcomes. Vinod [13] reported patients with BMI 24 or less are at significantly increased risk of in-hospital and long-term mortality after cardiac valvular surgery. This high-risk patient population warrants careful risk stratification and options for less-invasive valve therapies. Robert [14] found Increasing BMI has no independent association with worsened outcomes in the short or long term, and overweight patients have a survival benefit after surgery. So the relationship of BMI and long-term survival after AVR was controversial. The long-term survival research about BMI in small aortic root is rarely reported.

### Impact of BMI on long-term mortality and NYHA classification

It is important to note that there may be some differences in AVR between China and Western countries; it is unknown whether this could affect the results of the research. For example, rheumatic disease accounted for the major etiology in this cohort although occurrence of degeneration was on the rise; mechanical valves were mostly used; and patients were younger and had less concomitant CABG.

In this study, we determined that obesity raised the long-term mortality, implying that higher BMI means higher risk of adverse outcomes for patients with small aortic prosthesis replacement. These results are consistent with some previous studies [11,12]. In addition, a higher proportion of patients with poor cardiac function were observed in the obesity group at one year after surgery, and that distinction increased over time. These findings suggest that high BMI is a possible indicator of poor long-term quality of life for patients with small aortic roots. Numerous earlier studies [15,16] concluded that NYHA class of the patients could be ameliorated within a short period after operation. Nevertheless those studies they failed to keep up with its change in subsequent years after AVR.

### The long-term outcome of EOAI, LVEF, LVMI

Several factors such as EOAI, LVEF and LVMI may directly contribute to a higher class of NHYA class [17,18] in obesity and overweight group. We expelled LVEF from the list due to its insignificant change along with BMI increase. In contrast, EOAI and LVMI turned out to be related with BMI in our investigation. As a result, EOAI may associate with cardiac function and influence postoperative life quality. We also found EOAI to be also an independent predictor of late mortality in this research, which was in accordance with previous researches [17-20]. EOAI ≤ 0.85 cm^2^/m^2^ (considered as PPM) [21] could lead to increased late mortality after AVR, and that’s why PPM should be avoided in patients with small aortic roots. It’s a challenge for surgeons to select the optimal type and size of prosthesis, so that proper EOAI could be maintained in obese patients. Although suprannular stentless valves have been applied in clinic recent years to increase EOAI and prevent PPM, there have been some discrepancies in the results [22-27]. Annulus enlarging techniques, including Nicks procedure [1], the Manouguian technique [2] and the Konno procedure [3], allow for the implantation of prosthetic valves 1 or 2 size larger than the original size of the aortic annulus [28], and many studies have been frequently reported with good results, but some authors have reported increased operative mortality [28], so there is still no agreement about annulus enlarging techniques.

### Clinical implications

Why does obesity affect the late mortality and NYHA functional class in patients with implantation of small aortic prosthesis? We attempted to throw light upon the question in the following aspects: obesity-related diseases, operation techniques and postoperative lifestyle. Being overweight or obese were associated with increased risk of underlying disease such as diabetes [29], hypertension [30], coronary artery disease [31,32] and other chronic diseases [33-35], which may increase the mortality in the long-term. We indicated in this research that diabetes, systemic hypertension and chronic lung disease were independent predictors of late morality in patients undergoing AVR with small prosthesis. So obesity-related disease should be taken into consideration in advance to evaluate risk of surgery and predict long-term outcomes for the patients.

According to the results in our study, we should reconsider the role of annulus-enlarging techniques even though they are often accompanied by increased morbidity and mortality after AVR. Such techniques may be meaningful to recommend as the best choice for the patients with obesity and relatively small aortic annulus requiring AVR, but implantation with small prosthesis, especially in younger patients [36], can effectively increase the EOAI and improve the long-term outcome and life quality. So in the future we should work to perfect annulus-enlarging techniques, including reducing the cross-clamp time and the occurrence of complication.

It should be noted that in normal-weight group the EOAI and LVMI also worsened three years later in spite of lower later mortality. The main reason may be an increase in BSA. In this study, we were informed that many people gained weight after AVR due to being less active and an improper diet. Since height changed little after operation, accumulation of weight may bring about larger BSA. It is important to instruct patients with small prosthesis to keep fit and control weight, which could certainly benefit their health after AVR.

## Conclusions

This study analyzed the effects of BMI on the late mortality in patients undergoing AVR with small prosthesis. Results suggested that obesity was associated with increased late mortality after AVR in patients with implantation of small aortic prosthesis (size ≤ 21). Obesity and being overweight may also affect the NYHA function in the long term. EOAI should be improved where possible as it may reduce late mortality and improve life quality in such patients.

## Abbreviations

AVR: Aortic valve replacement; BMI: Body mass index; NYHA: New York Heart Association; LVEF: Left ventricular ejection fraction; EOAI: Effective orifice area index; LVMI: Left ventricular mass index; PPM: Patient-prosthesis mismatch; AF: Atrial fibrillation; CABG: Coronary artery bypass graft; CPB: Cardiopulmonary bypass.

## Competing interests

The authors declare that they have no competing interests.

## Authors’ contributions

This study was designed by BW and SW. BW and HY drafted the manuscript. All authors participated in the Data collection and compilation. GC and HY performed the statistical analysis. All authors read and approved the final manuscript.
